# The Role of PARP1 and PAR in ATP-Independent Nucleosome Reorganisation during the DNA Damage Response

**DOI:** 10.3390/genes14010112

**Published:** 2022-12-30

**Authors:** Ekaterina A. Belousova, Olga I. Lavrik

**Affiliations:** Institute of Chemical Biology and Fundamental Medicine, Siberian Branch of Russian Academy of Science, Novosibirsk 630090, Russia

**Keywords:** nucleosome core particle, DNA damage response, PARP1, PAR, histone H1, HMGB1

## Abstract

The functioning of the eukaryotic cell genome is mediated by sophisticated protein-nucleic-acid complexes, whose minimal structural unit is the nucleosome. After the damage to genomic DNA, repair proteins need to gain access directly to the lesion; therefore, the initiation of the DNA damage response inevitably leads to local chromatin reorganisation. This review focuses on the possible involvement of PARP1, as well as proteins acting nucleosome compaction, linker histone H1 and non-histone chromatin protein HMGB1. The polymer of ADP-ribose is considered the main regulator during the development of the DNA damage response and in the course of assembly of the correct repair complex.

## 1. Introduction

The eukaryotic genome consists of several billion pairs of nucleotides packed inside the micron-scale nucleus. The compaction degree of certain parts of the genome depends on both the stage of organism development and the type of differentiated cell [1]. In general, chromatin states can be classified into actively transcribed, i.e., euchromatin (form A), and its compacted form: heterochromatin (form B). In any case, 75–90% of genomic DNA is represented by a minimum unit of compaction: nucleosomes [2]. According to crystallographic data, the nucleosome is a 147 bp DNA duplex, left-handedly wound around the histone core [3]. The core is formed by four pairs of histones—H2A, H2B, H3, and H4—which form two dimers H2A-H2B and H3-H4. The length of DNA regions located between nucleosomes, i.e., of the so-called linker DNA, can vary from 20 to 350 bp, and this DNA can be bound to histone proteins. In mammalian cells, this binding is mainly performed by various forms of linker histone H1 [4].

Nucleosome core stability varies by 2–4 kcal mol^−1^ depending on the flexibility of DNA sequence [5]. This parameter is influenced by different factors such as poly(dA:dT) tracts, the CG content, the occurrence of YR dinucleotide steps, and epigenetic modifications, for instance cytosine methylation [6,7,8,9].

Approximately one-third of each histone protein consists of the unstructured regions, mostly located in tail regions and protruding outside the nucleosomal core. These segments play a substantial role in the stabilisation and mobility of nucleosomes both owing to a network of contacts with DNA and to protein-protein interactions with various nucleic-acid metabolism factors, for instance during transcription or DNA repair. Being highly evolutionarily conserved, histones are a hot spot for introduction of many functionally relevant modifications that could influence genome compaction. These include post-translational modifications (PTMs) [10,11] and sequence alterations based on of histone variants, isoforms, or mutations.

Another important characteristic of an actively transcribed genome region (e.g., a promoter region) is the density and pattern of the nucleosomes’ positioning. It has been shown that promoters of translation apparatus genes and of broadly expressed genes are characterised by a certain ordered nucleosome configuration and sufficient bendability that allows the formation of the correct transcriptional complex [12]. Global chromatin reorganisation requires the involvement of ATP-dependent remodellers. Nonetheless, non-histone DNA-binding factors can alter nucleosome positions through non-sequence-specific binding to nucleosomes; this phenomenon could lead to destabilisation or displacement of a nucleosome [13,14,15].

For correct functioning of the genome, the genome compaction degree should have certain variability that gives enough time—for example, during the transcription, or in response to DNA damage—to relevant proteins to access specific areas of genomic DNA. Recently, more and more data were published concerning the influence of various factors on the chromatin compaction degree. These factors include the action of ATP-dependent remodellers (ALC1, CHD1, ISWI, and SWI/SNF [16]), the participation of histone modification systems (e.g., acetylase/deacetylases and methylases) and interactions with non-histone proteins of the high-mobility group and with protein PARP1 [17].

Currently, there is a large amount of data regarding PARP1 and the poly(ADP-ribosyl)ation (PARylation) as one of the ATP-independent factors that influence chromatin remodelling. This review summarizes the basic data on this topic.

## 2. The Structure of the Nucleosome Core Particle (NCP) and Its Subtypes

The basic structural unit of chromatin is the nucleosome, the existence of which makes it possible to compact (shorten) linear genomic DNA by about 7 times. Interaction of nucleosomes with each other through linker histones or non-histone chromatin proteins provides a greater degree of compaction. Such plasticity of the genetic material is also necessary for cell development and differentiation, as well as for responses to changes in environmental conditions. In addition, a specific nucleosomal pattern is extremely important for the recognition of promoter regions of ubiquitously expressed genes by some proteins [12].

**Figure 1:**
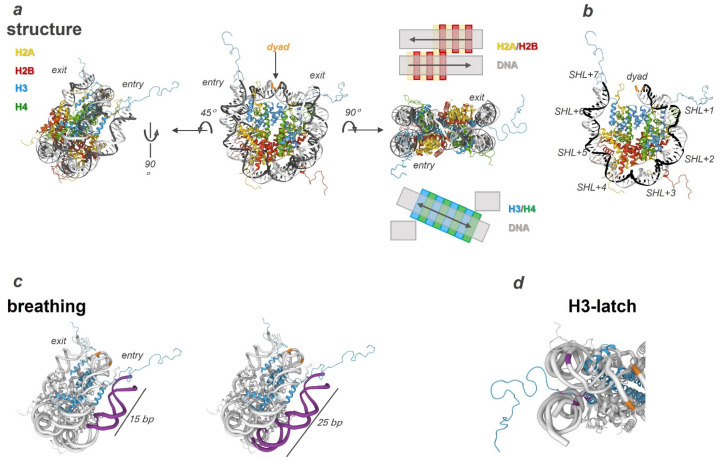
NCP structure and the key compaction factors. (**a**) The following elements of the core particle are highlighted in the image (Protein Data Bank (PDB) ID: 1KX5): DNA strands are grey and white, histones H2A are yellow, H2B is red, H3 is blue, H4 is green, and the dyad base pair is orange. All components are shown in cartoon representation. The side opposite to the dyad (bottom panel). The middle part is schematic representation of the histone dimer’s location relative to the DNA duplex in the NCP structure on the dyad side (upper panel) and on the opposite side (lower panel). (**b**) Cartoon representation of approximately half of nucleosomal DNA, in which superhelical locations are indicated clockwise in DNA from the dyad base pair to the duplex end. (**c**) The spontaneous fluctuations of nucleosome DNA gyres affect the compaction degree as seen on the DNA side [23]. (**d**) Specific contacts of the N-tail of H3 with DNA near the entry/exit site, a so-called latch. The amino acid residues and heterocyclic bases involved in the interaction are highlighted in purple, histone H3 in blue, the dyad in orange, and the other components in white [23].

NCP structure is relatively invariant among Metazoan [18,19]. In 1997, it was found that the NCP has a second-order symmetry axis that passes through a certain base pair of the DNA double helix [20]. This pair was named a dyad. Accordingly, 147 bp is the length of double-stranded DNA (dsDNA) within an NCP (Figure 1a). The DNA double helix turns going clockwise or counter-clockwise from the dyad are respectively denoted as +SHL or -SHL (superhelical location): from position 0 to position 7 [3,21] (Figure 1b). Thus, the histone core forms more than 120 contacts directly with all 14 SHL of dsDNA. The vast majority of them are mediated by the amino acid interactions with phosphate groups of the minor groove of the DNA helix. Additional contacts are based on interactions between Arg or Lys of histone tails and atoms of heterocyclic bases of the dsDNA minor groove [20,22,23]. Such interactions ensure that within an NCP, DNA double-helix geometry is very different from the classic B-conformation; this arrangement ensures the correct recognition and positioning of many factors of nucleic-acid metabolism [24,25,26,27,28,29].

Structural dynamics of the histone core and the corresponding density of dsDNA winding on the histone octamer determine the plasticity of the nucleosomal particle. Using the SELEX technology, Widom’s group identified certain sequences that give the highest stability in terms of the histone core and result in the assembly of the stablest nucleosomal particles: clones 601, 603 and 605 [30]. Subsequent in vitro experiments, bioinformatic analyse and numerous structural studies have characterised such regions of nucleosomal DNA in terms of the CG content, TA dinucleotides, and CA-TG steps [5,6,30,31,32,33]. In fact, a DNA sequence must have certain bendability in order to wrap around the octamer and at the same time to form a certain network of contacts with proteins; this property ensures the stability of the particle [34,35]. It has been found that the Widom sequence contains key elements—the so-called AT joints (AA/TT, TA and AT base steps)—located in the minor groove and oriented in a certain way towards the octamer, allowing unambiguous positioning of DNA relative to the histone core. GG, GC, and CG base steps should be shifted relative to more flexible AT by half a turn of the DNA helix [36].

Indeed, the CG content of NCPs has been shown to inversely correlate with the ability of nucleosomal DNA to support spontaneous particle unwinding [37,38]. It can be assumed that AT-rich regions are more often found near a transcription start site because they form less stable NCPs. Nonetheless, in *Homo sapiens*, the number of AT-less promoters exceeds the number of CG-less ones, and CG-based promoters have the highest prevalence (37.59% of all promoters) and are usually located in with housekeeping genes lacking a TATA box [39].

As already mentioned above, the nucleosomal pattern is essential for the correct positioning of general transcription factors and for transcription initiation. On the other hand, the presence of several NCPs near a transcription start site blocks the process of ongoing inappropriate transcription, and RNA pol II requires the presence of specific remodellers for the initiation for its transcription process. This means that the plasticity of the nucleosomal core plays an extremely important role in the stability of the NCP and in its ability to slide along DNA (reviewed in [15]).

The density of DNA duplex winding on the histone core varies and depends on how far a helix turn is from the dyad [40]. A study on thermodynamic stability of the nucleosomal core by Widom’s group indicates greater accessibility of a 10–12 bp DNA segment located in the region of the entry-exit site as compared to the dsDNA segments closer to the dyad [41]. In addition, Widom and colleagues have estimated the time during which the nucleosome stays in a completely wound state and duration of the state featuring partial loss of protein-nucleic-acid contacts of this NCP region: ~250 and 10–50 ms, respectively [31]. Today, these spontaneous fluctuations of the nucleosome compaction degree are called nucleosome breathing (Figure 1c). The data from Widom’s group points to a site exposure mechanism that may participate in nucleosome mobility and explains the access of nucleic-acid metabolism proteins to such a compact and fundamental structure as the nucleosome.

Indeed, a large amount of experimental data suggests that in vivo, approximately 50% of the nucleosomal pattern is specified by and explained by the genome’s primary structure and has characteristic features identified in in vitro experiments [36], reviewed in [9]. Nevertheless, the DNA sequence alone—i.e., the pattern of key protein-nucleic-acid interactions, which has been determined (among other things) from X-ray diffraction data—does not explain the results of in vivo experiments, indicating the plasticity of the histone octamer [42,43]. Currently, nucleosomes are considered not static but dynamic structures owing to structural alterations of the histone octamer in an ATP-dependent and ATP-independent manner that underline the dynamics of the regulation of genome-associated activities [44,45,46].

Relatively recently, Widom’s site exposure model was refined by molecular dynamics simulations. Namely, at the atomic level, Shaitan’s group presented functional modes of nucleosome dynamics such as spontaneous nucleosomal DNA breathing, unwrapping, twisting and sliding mediated by nucleosome core plasticity [23]. Those authors demonstrated that the ends of nucleosomal dsDNA are capable of rapid fluctuations by themselves on a time scale of 10–100 ns. At the same time, the kinetics of NCP unfolding/breathing take place in the microsecond range and are implemented precisely due to a conformational rearrangement of histone tails protruding outside the nucleosomal core [47]. Here, a special role is played by a smaller number and relatively low stability of protein-nucleic-acid contacts formed by the H2A-H2B dimer and by the outer half-turn of the dsDNA helix as compared to H3-H4. A considerable contribution to NCP stability is made by multiple interactions of the H3 αN-helix and of a nearby segment of the H3 histone tail (in particular H3Y41, H3R42, and H3T45 and the region between residues H3H39 and H3R49) with two stands of a DNA duplex. These contacts generate a kind of a latch insuring direct juxtaposition of two gyres of the DNA helix: by the positioning at nucleotide -9 near the dyad and at nucleotide 71 at the end of the nucleosomal DNA (Figure 1d) [23]. The interaction of the H3 tail with DNA results in less sliding of the nucleosome and stabilises interactions with the H2A-H2B dimer [48,49]. According to the refined model, NCP breathing affects unwinding up to the first 15 bp and proceeds within nanoseconds (~40 ns), whereas further unwinding leads to the loss of protein-nucleic-acid contacts for a 25 bp regions, i.e., latch; this loss occurs within microseconds. Due to the existence of local overtwisting and stretching of nucleosomal DNA, these more mobile 25 bp can be quite effectively pulled up to the dyad region [50,51]. In addition, DNA unfolding within the NCP is associated with local distortions of the DNA helix near the dyad (±SHL 1.0–1.5) and contributes to the loss of histone-DNA contacts in this region, and this loss in turn probably facilitates nucleosome sliding. Thus, at this time point nucleosome core plasticity is sufficient to ensure the variability of conformational dynamics polynucleosomal chromatin regions. It is possible that ATP-dependent remodelling proteins use this twist defect propagation to facilitate access to a lesion near the dyad during the repair process [31,32,52].

## 3. Histone Variants

Because the main role in the formation of the nucleosomal particle and in its plasticity is played by core histones, the existence of their variants and all kinds of PTMs, should significantly affect the genome compaction degree and accessibility dynamics of certain DNA regions. Controlling the variation of these parameters is important for successful interaction of transcription factors and repair complexes with various genome regions and must be dependent on the phase of the cell cycle and on an adaptive response. Variants of histones are known to be actively recruited to sites of DNA damage [53,54]. In addition, the nucleosomal core histones harbour more than 100 different PTMs, most of which are in N-terminal regions [10]. On the one hand, such specificity can promote chromatin decompaction at the site of DNA damage and trigger an appropriate repair pathway. On the other hand (for example, due to a PTM), histone variants can control NCP stability during repair in some phase of the cell cycle and can prevent further degradation of genomic DNA until it is completely restored, and these properties are especially needed in the case of double-strand break (DSB) repair. Below, the data on the involvement of the main variants and modifications of core histones will be briefly summarized for the repair process in response to the emergence of damage in genomic DNA.

Core histone H2A is the most “mobile” element of the nucleosome. Variants of this histone vary in the length of C- and N-tails, which has a major effect on the formation and stability of the nucleosome particle [55]. Many cancer types are associated with an alteration in the terminal region of H2A variants [56]. The presence of the best-characterised H2A.B variant in the genome causes the formation of a non-canonical form of the NCP at position~118 bp and increases cell sensitivity to the action of DNA-damaging agents [57,58]. Variants H2A.X, H2A.Z and macroH2A are participants in various DNA damage response (DDR) pathways [59]. In human cells, the H2A.Z variant is recruited to DSB sites thereby resulting in the assembly of homologous recombination (MRE11, BRCA1, and RAD51) and non-homologous end-joining (NHEJ) (proteins KU70 and KU80) complexes [60]. In addition, there is evidence of the importance of H2A.Z in the mismatch repair (MMR), base excision repair (BER), and nucleotide excision repair (NER) processes [61,62].

The H2A.X variant is a mark of DSBs in DNA. On the one hand, the presence of the H2A.X in a nucleosome is the main target for a PTM (mainly at pS139), which is catalysed by a different class of kinases and is crucial for the initiation and regulation of the correct DSB repair pathway for a DSB [63,64]. On the other hand, the presence of (y)H2A.X in nucleosomes after interaction with PARP1 (the sensor of DNA single-strand breaks), increases the association rate and stability of the entire complex and enhances the catalytic activity of PARP1, which is necessary to start the repair process [65].

It is known that macro-domains in proteins are responsible for the binding of poly(ADP-ribose) (PAR) [66]. MacroH2A histone variants also play a considerable role in the initiation of the repair processes associated with PARP1 activity—NHEJ, homologous recombination (HR) and BER. The macroH2A1.1 variant is indeed capable of binding the PAR synthesised by PARP1 in response to oxidative stress. A histone is recruited to the DSB by binding to the PAR attached to PARP1 after the latter it is relocated to the damage site, rather than being directly recruited to a DSB as part of the nucleosome [67,68]. Such macroH2A1.1 binding affects the kinetics of PAR accumulation and as a consequence leads to an increase in the lifetime of the polymer and to the suppression of PARP1 activity [69]. The presence of macroH2A1.1 promotes CHEK2 activation and recruitment of NHEJ proteins KU70/80 and 53BP1 to the damage site [67]. For instance, the presence of the macroH2A1.1 variant in an NCP promotes effective repair affecting the NAD^+^ pool (and its maintenance) in the cell under oxidative stress. Nevertheless, another common form of macroH2A1, macroH2A1.2, is formed by alternative splicing that removes key residues in the macrodomain responsible for the recognition and binding of PAR [70]. Recruitment of macroH2A1.2 to DNA breaks is independent of PARP1 [71,72]. In any case, the activity of BER is more effective on NCPs containing macroH2A variants [61,67,69].

The second component of the dimer, histone H2B (or rather its phosphorylated and ubiquitinylated forms), appears in the NCP within an hour after the action of the agents responsible for DSB formation and contributes to effective recruitment of HR factors—BRCA1, CtIP, and NBS1 [73,74,75,76].

The second H3-H4 dimer is the most stable component of the NCP and binds to DNA immediately after its synthesis [77]. One of the beat-studied variants of H3 CENP-A is a component of centromeric nucleosomes and determines the position of kinetochores in the course of chromosome segregation during cell division [78]. On the other hand, another variant H3.3, is considered necessary for PARP1-dependent NHEJ [79]. In addition, PTMs in the H3 latch—H3Y41 and H3T45 phosphorylation (which is important for the maintenance of electrostatic interactions with DNA) and H3R42 methylation (which is needed for the propensity of H3 to interact with the DNA minor groove) can significantly affect the stability of interactions between the histone core within the NCP and facilitate access to a lesion at a distance from the entry-exit site [23]. Multiple PTMs of the canonical H4 variant determined the choice of a repair pathway after a DSB emerges, e.g., via their influence on chromatin dynamics [80,81,82,83].

## 4. Linker Histone H1 as a Factor Affecting Chromatin Compaction Dynamics

By the end of the 20th century, the 166 bp dsDNA associated with the octamer and with histone H1 had been isolated within the structure of chromatin and named chromatosomes [84]. This non-core histone binds to DNA linker regions near the entry-exit site and strongly alters conformational dynamics of the NCP, and simultaneously, the compaction of chromatin [4,85] (see below).

In mammalian cells, there are 11 variants of H1, and the H1.0 variant is characteristic of non-dividing and terminally differentiated cells [86]. All proteins of the H1 family undergo several types of PTM, including phosphorylation, methylation, and acetylation, with phosphorylation being especially common; the extent of this modification increases during interphase [87,88,89]. Expression levels and distribution of H1 variants influence the cellular phenotype and terminal differentiation [90,91]. In general, the expression of all H1 genes is controlled at transcriptional, post-transcriptional and post-translational levels. In addition, the H1 protein level in cells of high eukaryotes varies greatly (from 0.4 to 1.0 molecule per NCP, rarely reaching 1.0), but an increase of this ratio above 1.0 can cause the appearance of two H1 molecules in the NCP and a decrease in the density of the local nucleosome pattern [92,93]. Because of such a wide variety of forms and owing to the specificity of their distribution across the genome during development and cell differentiation, all of them should differ in binding affinity. In addition, this characteristic has to be dynamic [94].

All proteins of the H1 family have a characteristic tripartite structure, in which a conserved globular domain (GD, ~80 aa) and the surrounding domains [short N-terminal (NTD, 13–40 aa) and longer C-terminal domains (CTD, ~100 aa)] can be distinguished [95]. These are lysine-rich and most positively charged proteins of eukaryotic cells [95,96]. Both termini are highly variable and undergo multiple PTMs [89,97].

**Figure 2:**
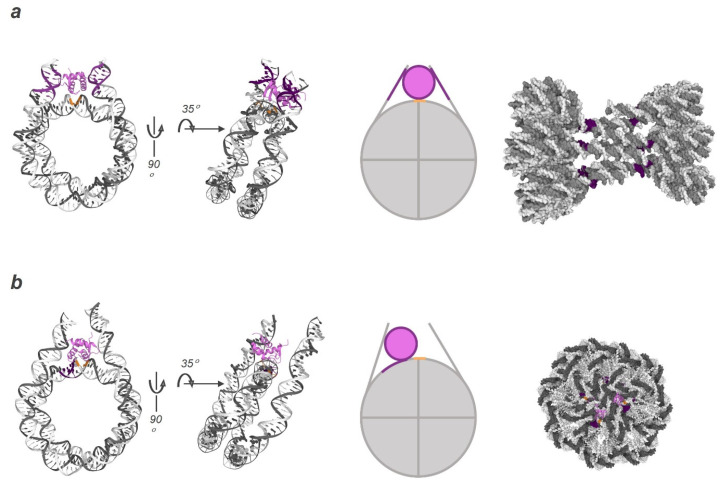
Chromatosome structure: the on- and off-dyad binding model. Cartoon representation of nucleosomal DNA (in grey and white) and a linker histone (purple). The DNA positions juxtaposed to the protein are highlighted in magenta, and the dyad in orange. Core histones are not shown for clarity. (**a**) Nuclear magnetic resonance structure of the NCP core with 167 bp DNA based on Widom’s “601” sequence in complex with the globular domain (GD) of chicken linker histone H5 (PDB ID: 4QLC). The linker histone is engaged in stable symmetric interactions with the dyad and both DNA linkers. Such interactions induce the formation of a ladder-like conformation of the chromatin fibre. (**b**) Nuclear magnetic resonance structure of the NCP core with 197 bp DNA based on Widom’s “601” sequence in complex with human linker histone H1.4 (PDB ID: 7PFD). The linker histone is displaced from the dyad position towards one of the linker regions. This interaction is affected by the local nucleosome environment and leads to twisted geometry of the fibre conformation.

The binding of H1 to the NCP is quite well described. The primary determinant of the type of H1 binding to the NCP is a highly disordered C-terminal domain [98,99]. There are two main models, which are referred to as on- and off-dyad judging by the histone location relative to the dyad (Figure 2). According to the first model, H1 is positioned directly along the axis and interacts with ~10 bp of the DNA duplex minor groove of both NCP linker regions [100] (Figure 2a). In this case, chromatin is compacted into a structure with a zig-zag arrangement of NCPs relative to each other, similar to a ladder, which leads to a loss of packing of NCPs meaning greater accessibility of genomic DNA [101]. In the off-dyad model, H1 is in a conformation predominantly interacting with one of the linker regions, and the globular domain is situated in the major groove of the DNA duplex with a 3–7 bp offset relative to the dyad (SHL approximately +0.5); this situation leads, first of all, to the restriction of free breathing of DNA gyres, and next to the formation of denser fibres and to consequent lower accessibility of the genomic DNA [102,103,104] (Figure 2b). The implementation of the latter chromatosome conformation implies competitive interactions for example, between a linker histone (and some isoforms of core histone H2A) with an occluded part of DNA, thereby causing transition from the compacted form to the unwrapping of ∼10–15 bp at each end of the NCP [101]. Moreover, these two conformations exist in a dynamic equilibrium, which may be related to a change in the H1 binding configuration and shifted by slight alterations of the ionic environment and interactions with the H3 tail of the NCP core [105,106,107,108].

Today, there is evidence that H1 binding is involved in the formation of eu- and heterocompartments and largely determines the accessibility of chromatin because transcriptionally active and regulatory intergenic regions are depleted in H1 [109]. Nonetheless, the binding of H1 to genomic DNA is not a stochastic process. It has been found that the lifetime of H1 in chromatin is several minutes, while the rate of core histones’ exchange is in the hourly range [94,110]. Fluorescence recovery after photobleaching experiments has also revealed the dependence of the H1 exchange rate on the functional state of chromatin: the exchange rate is higher in transcriptionally active chromatin that in inactive chromatin [110]. These data suggest that, if necessary, H1 can be replaced by an alternative protein that somehow affects the chromatin compaction degree during some process [111,112]. Proteins’ competition for binding sites near the NCP is a component of the general mechanism that ensures functional and structural plasticity of chromatin fibres [113].

Indeed, in a study on H1 partners inside the cell, a large number of proteins were identified that are responsible for various cellular processes related to the maintenance and reproduction of genetic information [114]. Among them, there are repair-regulatory proteins Ku70/80, DNA-PK, YB1 and PARP1 [115,116,117,118]. On the one hand, the presence of structurally disordered domains in H1 should yield a large repertoire of protein–protein interactions. On the other hand, despite the participation of the structured globular domain in protein-protein interactions, its well-defined interaction with nucleosomal DNA binds H1 to chromatin [115]. It is possible that in the constantly changing nuclear environment of mammalian cells, owing the network of protein-protein interactions, H1 plays the role of a hub and is involved not only in the formation of a specific structure but also in functional control [119].

Therefore, conformational dynamics of linker DNA can be regulated by H1 binding mode and by the immediate environment of the NCP. In addition, a change in the stoichiometric ratio of H1 and to NCP can significantly affect both the architecture of chromatin and its local dynamics in mammalian cells. The ability of H1 to interact not only with NCPs but also with many proteins, e.g., during the development of the DDR, indicates direct involvement of the reorganisation of certain chromatin regions in a specific way, for example, for a repair process.

## 5. HMGB1 as an ATP-Independent Chromatin Remodelling Factor

The degree of chromatin compaction is affected by both histones and non-histone proteins. These include, among others, abundant nuclear protein HMGB1 (high mobility group box protein 1): a small protein affiliated with the high-mobility group (~25 kDa). The HMGB1 amount is estimated as 10^6^ per cell, which is ~10 times less than the amount of H1 [120,121]. This protein is associated with many biological processes including the regulation of chromatin structure, transcription and the DDR [122,123].

The unicity of HMGB1 also lies in the fact that it is possibly the most conserved protein among mammalian ones: it has only two substitutions out of 214 amino acid residues in the primary structure of the protein [124]. Furthermore, HMGB1 binds quite weakly to the B-form of DNA compared to its alternative forms and does so almost sequence-independently [123,125]. HMGB1 has three structural domains, two of which the N-terminal Box A and central Box B are basic domains, and the C-terminal one has an acidic tail; the basic linker regions are located as follows: one between two boxes, and the other between the boxes and the acidic tail. Boxes A and B share up to ~30% identity in primary structure, and the C-terminal domain contains ~30 alternating aspartates and glutamates [126]. Despite the great similarity between the boxes, the main DNA-binding activity is mediated by the A box, whereas the B box is mainly responsible for pro-inflammatory activity [127,128]. There is evidence that the C-terminal domain is involved both in the process of DNA binding and in the regulation of DNA damage repair [129,130].

**Figure 3:**
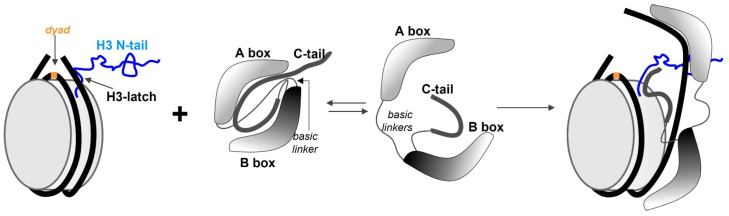
The interaction of HMGB1 with the NCP. Schematic representation of nucleosomal DNA (black), a histone octamer (a grey cylinder), HMGB1 (A and B boxes, two basic linkers and the acidic C-tail) and the basic N-tail of H3. The dyad is highlighted in orange. In the absence of DNA, HMGB1 is in a closed conformation that is transformed by non-sequence-specific interaction with linker DNA at the entry/exit site of NCP. The complex is stabilised by the interaction between the acidic tail of HMGB1 and N- tail of H3; the interaction drives DNA unwrapping and NCP destabilisation.

HMGB1 interacts with the NCP near the entry-exit site near the N-terminus of H3 between two gyres of the DNA helix [131,132,133] (Figure 3). Lysine and arginine residues, which are distributed evenly throughout the two arms of HMGB1, and aromatic amino acids interact with the dsDNA in the minor groove. Such interplay disrupts the system of van der Waals, electrostatic, and partially hydrophobic interactions within the NCP and leads to a loss of DNA rigidity and to bending of the DNA duplex in the direction of the major groove by slightly more than 60°, which varies from 80° to 140° for different proteins of the HMGB group) [127,134]. It has been shown that the interaction of HMGB proteins with the mononucleosome causes local ATP-independent structural changes that are not associated with sliding, thus providing greater access to the dyad region and to the periphery of the nucleosome core region [135]. This phenomenon is reflected, in particular, in the several-fold enhancement of the affinity of site-specific binding proteins for restructured NCPs [136]. The mechanism by which the reorganisation of the NCP proceeds is not linked to the simple unwinding of DNA relative to the core [137,138]. The interaction of HMGB1 with the NCP leads to the emergence appearance of two subpopulations of restructured nucleosomes having distinct conformations that differ in physical parameters from the structure of the canonical particle. Of note, that reorganised NCPs are stable even after protein dissociation [139]. It is thought that an important role in the interactions of HMGB1 with the NCP is played by the C-terminal domain, whose competitive binding to the H3 N-tails introduces a partial loss of contacts between the N-tails of H3 and H4 and dsDNA within the NCP (Figure 3) [140,141]. Nonetheless, stability studies on restructured NCP containing different truncated core histones in vitro suggest that the effect of HMGB1 is more complicated and influences various forces within the nucleosome [139].

The interaction of HMGB1 with the NCP is a very dynamic process and proceeds within the seconds’ [142]. After the restructuring, the final forms of the NCP remain sufficiently stable for tens of minutes and, although they are in equilibrium with the canonical form, they can revert to the original NCP under the action of various external factors [139].

Such a distortion of DNA structure and the disruption of major interactions (for example, those that hold the NCP together with the H3 tail) can give rise to a heterogeneous and dynamic population of NCPs, some of which can easily associate with various factors, including chromatin remodellers [138,143,144,145,146]. Besides, after NCP binding, the distortion of DNA structure, and recruitment of an appropriate transcription factor or remodeller, HMGB proteins can rather quickly dissociate from the ternary complex via the “hit and run” mechanism, thus performing the function of a chaperone [147]. Consequently, the interaction of an HMGB with chromatin promotes not only partial destabilisation of the NCP but also its ability to slide along DNA owing to ATP-dependent remodellers [124].

HMGB1 can influence the processes related to chromatin maintenance, for example, DNA repair, not only owing to a significant distortion of the substrate but also due to direct interactions with repair proteins [123,148]. Recent cell biological and biochemical research indicates that HMGB1 actively participates in the modulation of the efficiency of four major DNA repair pathways, i.e., NER, BER, MMR, and DSB repair, including NHEJ [137,146,148,149,150,151,152,153].

Another possible important feature of HMGB1 functioning in mammalian cells is indirect control of NCP assembly because HMGB1 deficiency leads to a deficiency of all types of histones and to subsequent depletion of the NCP pool [154]. The genome of such cells is extremely sensitive to DNA-damaging agents. Additionally, HMGB1-deficient cells show high transcriptional activity. Experiments in HeLa cells have revealed that the predominant nuclear localisation of HMGB1 in most cell types is a result of steady-state conditions in which HMGB1 molecules are constantly transferred from the cytoplasm to the nucleus and back by energy-driven transport processes [145].

Therefore, during interaction with the NCP, the non-histone chromatin protein HMGB1 may act as a dynamic alternative to the main linker histone H1 [122,155]. Competition between these proteins should result in the opening and closing of accessible domains on specific nucleosomes while maintaining the overall structure of chromatin. Furthermore, due to its wide network of protein-protein contacts, HMGB1 when recruited to damaged chromatin may help to stimulate an appropriate DDR pathway.

## 6. Nuclear Protein Poly(ADP-ribose)polymerase 1 (PARP1): Interaction with the NCP

Another important nuclear protein that actively interacts with chromatin is PARP1. It belongs to the poly(ADP-ribose)transferase family, which has 17 genes in the human genome [156]. A distinctive feature of this family of proteins is the presence of a unique H-Y-[EDQ] PARP-signature domain in the active site of the enzyme. Nonetheless, it is reported that not all members of this family are able to bind substrate NAD+ molecules and have catalytic activity. Moreover, only two members, PARP1 and PARP2, are nuclear DNA-activated proteins and synthesise the ADP-ribose polymer, whose covalent or non-covalent binding regulates many cellular processes including DDR initiation [156]. This is one of the reasons why PARP1 is called the key keeper of genomic stability [157,158]. The recruitment of a PARP1 molecule to the site of genomic DNA damage is one of the fastest cellular processes, and the interaction of PARP1 with chromatin itself and the resultant PARylation are involved in the regulation of chromatin dynamics, replication, cell cycle control, apoptosis, and other phenomena [159].

A large body of biochemical in vitro data gives an idea about the PARP1 interaction with free DNA and with the NCP. Towards DNA duplexes, the strongest affinity of PARP1 is observed in the presence of blunt ends or a 5′-phosphorylated single-strand break in DNA [160,161,162]. During the interaction with nucleosome particles, PARP1 shows consistent selectivity: initial complexes are formed by one PARP1 molecule located near blunt ends or near linker regions of nucleosomal DNA; after the binding of other molecules, PARP1 has additionally been found to be located near the entry-exit site [163,164,165,166]. It has been shown that PARP1 binding to an end of nucleosomal dsDNA leads to a significant increase in the distance between adjacent gyres of the duplex and this process is not accompanied by a loss of histones and is reversible after PARylation [167]. Thus, the interaction of PARP1 with the NCP in a cluster of nucleosomes drives the reorganisation of the nucleosome particle, and the magnitude of this reorganisation depends on local concentration of the protein molecules in question. At the low concentration of the protein, one PARP1 molecule binds and locally displaces one end of nucleosomal DNA from the surface of the histone octamer, whereas a higher concentration of the protein results in the combined action of two molecules of PARP1, leading to more extensive rearrangement of the nucleosome [163]. In this case, one of the PARP1 molecules binds to the NCP in an H1-like manner near the entry-exit site. In vitro data correlate with in vivo results, which indicate the reciprocal nature of the interaction of PARP1 and H1 with chromatin of promoters of genes actively transcribed by RNA pol II [111,168]. In addition, it has been shown that PARP1 binds rather quickly with strong affinity to the H2A.X-type nucleosome as compared to the H2A nucleosome; the time of half-accumulation is only 1.6 s [65,159,169]. In this regard, the association kinetics accelerated by the presence of the H2A.X variant may contribute to a key step in the repair process: the accumulation of PARP1 followed by partial chromatin reorganisation and PAR synthesis.

In the presence of NAD^+^, the binding of PARP1 to DNA, either free or as a part of the NCP, triggers PARylation with covalent attachment of a PAR molecule to an acceptor protein [170]. In this case, both PARP1 itself and the protein located in the vicinity of the binding site can serve as an acceptor [171]. All core and linker histones are reported to serve as PAR acceptors, albeit not the best ones among cellular proteins, and H1 has been found to be the best PAR acceptor among histones both in vitro and in chromatin, whereas in the response to DNA damage, H2B and H3 are the best PAR acceptors [172,173]. Relatively recently, histone PARylation factor 1 (HPF1 protein) was discovered, the presence of which causes dramatic redistribution of PARylation acceptors; this redistribution first of all targets this enzymatic modification to a different amino acid and sharply increases in the efficiency of the modification of histones, especially H3 [174,175].

**Figure 4:**
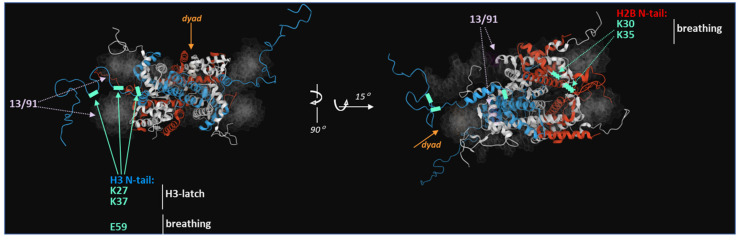
NCP contacts—involved in the stabilisation of NCP structure through the H3-latch and breathing—that could be potentially affected by PARP1 interaction and subsequent PARylation (data from [23,163,167]). Specific PARylation sites in these regions are highlighted in a mint colour and indicated by mint arrows (a dashed line for H2B contacts and a solid line for H3 contacts). DNA is blurred, and histones H2A and H4 are highlighted in white for clarity. The dyad is highlighted in orange. Dashed whitishe-pink arrows go to positions of the fluorescent dyes that were used for the investigation into the molecular dynamics of the NCP after PARP1 binding and PARylation [163,167].

The PAR molecule represents a long negatively charged polymer, and it is commonly thought that such a PTM leads to steric and electrostatic repulsion and therefore dissociation of the modified molecules from the complex with DNA. This principle applies to both histones and PARP1 itself [172,176] (Figure 4). Indeed, PARylation enhances the dissociation of PARP1 from DSB sites [65,165]. A research article about kinetics of PARP1 accumulation on and dissociation from the NCP in the presence of H2A.X leads to a conclusion that the presence of an alternative histone variant can promote either association or a final release of PARP1 after self PARylation during DNA repair [65]. In this context, the substrate, which is the partner in the PARP1 interaction, comes to the fore. For instance, it is reported that the presence of a mutant PARP1 having an impaired catalytic activity leads to an extremely weak reaction during the initiation of the DDR in mice [177]. Normally, the level of NAD^+^ in the cell is relatively high; however, during the first 15 min after DDR activation, its concentration decreases to 20%, and after 30 min, it drops to almost an undetectable level [169,178]. In addition, ATM-mediated phosphorylation of H2A.X generating γH2A.X occurs already after PARP1 relocation to the DNA damage site, and this localisation can persist for at least 30 min. In this sense, hyperactivation of PARP1 by γH2A.X-containing nucleosomes at DSB sites can give prolonged ADP-ribose synthesis in a situation when NAD^+^ concentration is extremely low [65].

In addition, PARP1- and PARylation-induced chromatin re-compaction has been demonstrated during transcription [179,180]. Studies on the regulation of pS2 promoter expression in MCF-7 cells have shown that after binding of appropriate transcription factor of the *ERE* promoter, the TopoIIβ-PARP1 complex is recruited, which inevitably induces sequential DNA cleavage and PARP1 activation [179]. The synthesis of PAR leads to simultaneous recruitment of HMGB1 or HMGB2 and a release of the previously bound H1 histone, to further changes in the local chromatin conformation and to transcription activation transcription [179]. A similar mechanism in the regulation of the transcription of other genes has been identified [180]. It is possible that such a pathway is more universal and implemented during the repair of the compacted form of DNA.

## 7. PAR in the DDR

One of the fastest and most comprehensive responses to damage in mammalian genomic DNA is PAR synthesis, 70–95% of which is catalysed by PARP1 [181,182]. Generally, it is thought that the length of this polymer does not exceed 200 units and that PAR can have linear and a branched structure [183]. Another variable that expands the PAR repertoire is the amino acid acceptor, to which the first ADP-ribose is covalently attached. Glutamate, aspartate, arginine, asparagine, lysine, cysteine, histidine, tyrosine, and serine/p-serine residues are among the main acceptors that have been identified so far [171,184,185,186,187,188,189,190].

**Figure 5:**
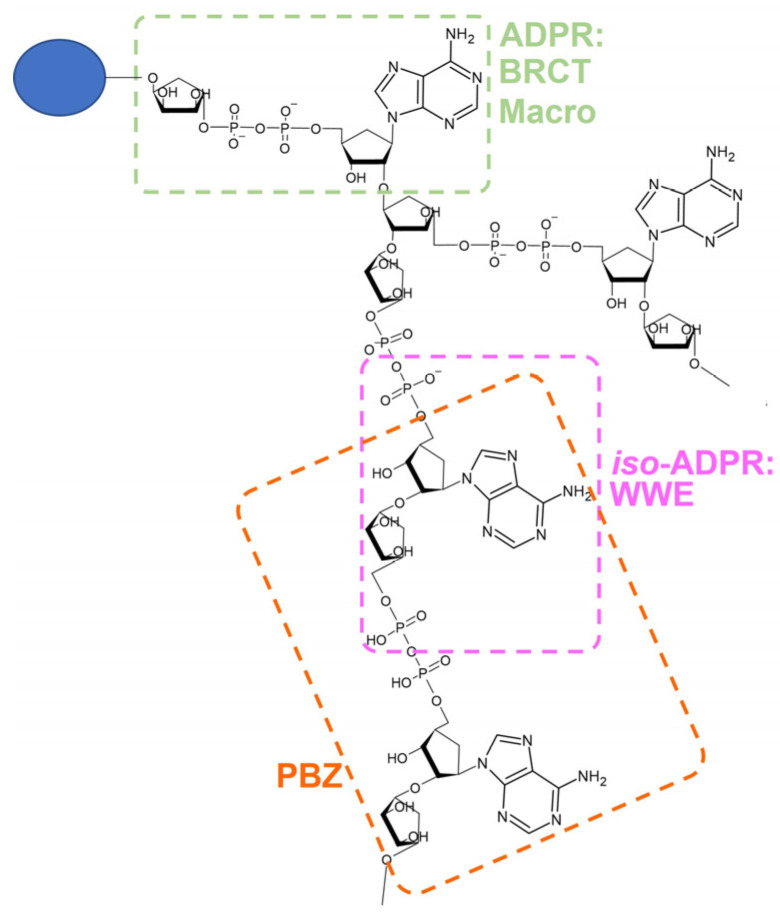
PAR structure and the specific motif affecting preferences of various protein domains.

PAR can influence protein-protein and protein-nucleic-acid interactions not only by being covalently attached to a target but also by engaging in a competitive interaction with other substrates owing to the presence of PAR-binding domains in proteins. Indeed, PAR effectors can recognise different sections of PAR chains (Figure 5). For example, iso-ADP-ribose (iso-ADPR) the smallest structural unit of a PAR chain and contains the ribose–glycosidic bond; specific recognition of iso-ADP-ribose is mediated by the oligonucleotide/oligosaccharide-binding (OB) fold (OB-fold) or by forkhead-associated (FHA) or by WWE (conserved tryptophans and glutamates) domains [191,192]. At the same time, recognition of the entire ADP-ribose units is implemented by macro or BRCT domain, and two adjacent ribose groups of PAR are recognised by PAR-binding ZnF (PBZ) domains [66,191,193,194].

**Figure 6:**
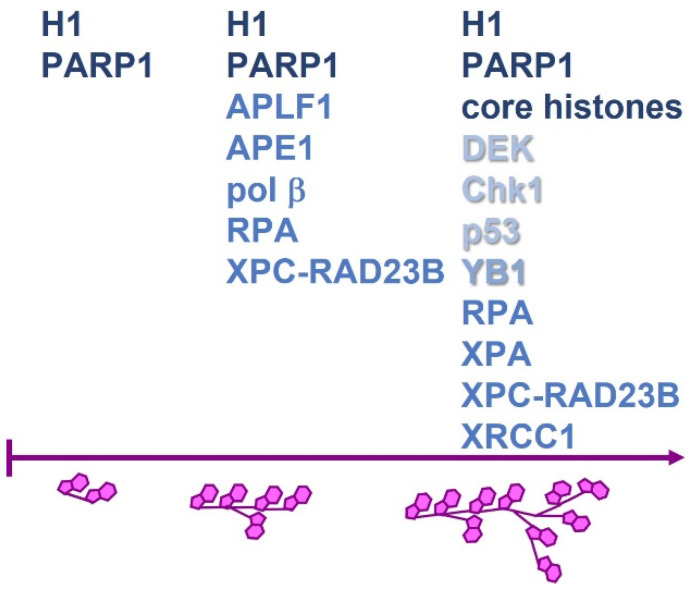
Affinity of various proteins towards different PAR chains. ADP-ribose units are represented by a magenta double-polygon. An increase in length and the number of branch points in the polymer correlated with the number of ADP-ribose units.

It is clear that the affinity of various proteins for PAR should be altered not only by the type of PAR-binding domain but also by the type of ADP-ribose polymer itself [195,196,197,198]. Indeed, it reported that different proteins could have an affinity for different types of PAR chains; short (10-mers), medium (20–30-mers, containing branching), and long (branched chains of more than 40–50 units of ADP-ribose in total); the affinity is in the range of 10^−9^ to 10^−6^ M [199] (Figure 6). It should be noted that the branching depends on the type of ARTD protein, and in case of PARP1, it occurs approximately once every 20–50 ADP-ribose units [200,201]. Such DDR factors as DEK, Chk1, XPA and p53 and DNA repair proteins RPA and XPC-RAD23B preferentially interact with long PAR chains, whereas BER proteins APE1 and Polb preferentially bind to a linear form of oligomeric PAR and medium-length PAR chains: 8- and 20-mers. On the other hand, NHEJ-specific histone chaperone APLF specifically recognises branch points [191,202,203,204,205,206,207,208]. Core histones preferentially bind to branched and longer PAR chains, whereas the linker histone H1 has the strongest affinity for and can bind to PARs of different lengths, even very short chains [203,206]. As for PARP1 itself, it is reported that as the length of PAR increases, so does the affinity of this protein for this polymer [209]. It is noteworthy that the affinity of the PARP1for PAR is the same range as the affinity of unmodified PARP1 for the NCP [65,163,165,210]. Therefore, during the interaction of PARP1 with damaged nucleosomal DNA, activation and subsequent self-modification of PARP1 should lead to an inevitable breakup of the PARP1-NCP complex owing to competitive interactions [176,210].

A heterogeneous population of PAR chains has been found in different tissues and cell types, thereby possibly supporting the theory about of a relation between the type of synthesised PAR and the signal leading to its appearance [197]. Indeed, a number of authors have identified associations between PAR chain length and protein partners during PARP1 activation in the course of DNA repair. For example, short and highly branched PAR is less effective in attracting XRCC1 to a damaged site [197,210,211]. On the other hand, the synthesis of a shorter PAR is observed in the presence of RPA, YB1, and HPF1 in vitro [174,198,208,212,213]. Moreover, these proteins are thought to regulate PARP1 retention time on DNA by reducing the length of PAR chains and/or by raising the efficiency of trans-ADP-ribosylation of histones in the context of cis-modification of PARP1 itself. As noted previously, auto-PARylation drives the dissociation of PARP1 from its complex with damaged DNA. It has been shown that the dissociation of PARP1 depends on the length and type of PAR branching. It has been revealed that the presence of shorter but more branched chains leads to the dissociation of the complex, apparently not only owing to electrostatic repulsion, but also because of a general steric effect [197]. This investigation into PARP1 mutants that are capable to synthesising various types of PAR chains and into their effect on cell physiology grave the authors (of the article just cited) the idea that PAR branching promotes chromatin remodelling during the DDR [197]. In general, more branching is registered during the catabolic phase of a genotoxic-stress-induced PARylation response in the cells; the recruitment of the NHEJ-specific APLF chaperone to the branch sites supports the theoretical model proposed in refs. [197,207].

The stability of the ADP-ribose polymer is another factor that is important for the dynamics of protein-nucleic-acid complexes in response to DNA damage. Because of the high structural diversity of PAR chains and of their molecular acceptors, PAR-cleaving enzymes must also constitute a large community. Indeed, the hydrolysis of ADP-ribose bonds is carried out by members of two evolutionarily distinct protein families related to macrodomains and (ADP-ribosyl)hydrolases (ARHs) [214]. The most abundant and widely specific enzyme of this class in mammals is PARG, which hydrolyses the ribose–ribose glycosidic bond but cannot act on the terminal protein–ribose bond [215]. ARH1 is responsible for the cleavage of the last residue attached to a target protein via arginine, whereas ARH3 possesses broader substrate specificity including recognition of a modified serine residue [216,217,218]. PAR turnover, i.e., the duration of its existence in the cell, amounts to seconds’ [219]. Nonetheless, long PAR molecules are cleaved by PAR-catabolising enzymes faster than shorter ones are [220]. In addition, PAR branching can lead to the stabilisation of PAR structure because PARG appears to prefer degrading of a linear part of the polymer over branching points [221,222]. It bears repeating that the branching occurs on average once every 20–50 ADP units during PARP1-catalysed synthesis [200,201].The finding that the protein fraction that binds to the longer polymer is enriched with factors of nucleic-acid metabolism including replication, mismatch DNA repair and splicing factors, supports the idea proposed in ref. [209].

Considering all of the above, it is likely that the duration of PARP1 accumulation at a DNA damage site and the time of activation of its catalytic activity are important for DDR regulation in the cell. This means that the cell needs the fastest possible transmission and implementation of this intracellular signal. On the one hand, this process contributes to rapid assembly of repair complexes at the accessible damage site thereby helping to preserve genome integrity. On the other hand, it preserves the NAD^+^ pool [65,159,169,177,178]. In response to DNA damage, PAR synthesis occurs quite quickly, within seconds. Accordingly, because of the diversity of polymer chain types, PAR can serve as a kind of scaffold for the formation of special intracellular biomolecule condensates [183]. The presence of specific PAR-binding domains should ensure the recruitment of specific proteins to the damage site followed by the formation of biomolecule condensates [179,180,183,223]. Timing of PAR length is controlled during the DDR, when long polymers (>22-mers) are rapidly synthesised by PARP1 and then are slowly degraded into shorter chains; consequently, it is possible that PAR length governs the dissociation of PARP1 from DNA and accordingly assembly of specific complexes [197,209].

## 8. Conclusions

Therefore, the various data accumulated in in vitro and in vivo experiments point to a direct role of PARP1 and PAR in the relation between (i) elements of genome plasticity, i.e., histones, and (ii)_ the involvement of specific repair proteins coordinating or implementing one or another pathway.

Numerous in vitro studies have shown that in compacted chromatin, linker histone H1 and non-histone chromatin protein HMGB1 compete with each other for interaction with the entry-exit site or linker DNA of the NCP [122,135,224]. After the emergence of a specific signal, the recruitment of PARP1 to the affected DNA site promotes the accumulation of PAR. Its structure is important not only for chromatin decompaction but also for the sequential assembly of certain protein complexes [225,226]. Accordingly, during the repair of compacted DNA, a release of the linker histone H1, re-compaction of chromatin with the help of non-histone proteins such as HMGB1 and HMGB2, and access to the lesion can be implemented by the rapid dynamic interaction of PARP1 with the autoribosylated form.

In any case, either direct binding of PARP1 to the NCP, whether H1-like or DNA-mediated, or recognition—by specific proteins—of PAR structure, whose synthesis was catalysed by PARP1 upon the interaction with damaged DNA, contributes to the destabilisation of the nucleosome particle and to the assembly of the correct repair complex [169,227,228]. Auto-PARylation of PARP1 leads to the dissociation of the complex, thus governing the dynamics of the repair process [65,165]. A possible driving force of the entire process can be the ADP-ribose polymer, and its structure can be determined by the type of damage and by DDR-triggering proteins. Disturbances in the system of PAR-mediated formation of biomolecule condensates have been implicated in the onset and progression of various pathological states, such as cancer, viral infections, and neurodegeneration; thus, control over the formation and dynamics of such condensates by means of a combination of PARP1 inhibitors may be key to the treatment of some human diseases [183,223].

## Data Availability

Not applicable.
